# Healthcare professionals’ perspectives on contextual factors related to (re)referral and (re)admission to geriatric psychiatry of people with dementia and behaviour that challenges living in nursing homes in Germany: a qualitative study

**DOI:** 10.1186/s12912-025-04117-2

**Published:** 2025-12-23

**Authors:** Imane Henni Rached, Victoria-Fabiola Ullmer

**Affiliations:** 1 University of Bremen, Bremen, Germany; 2 Rheinhessen-Fachklinik Alzey, Alzey, Germany; 3 Catholic University of Applied Sciences, Mainz, Germany

**Keywords:** Qualitative study, Contextual factors, Referral, Admission, Dementia, BPSD, Challenging behaviour, Crisis, Psychiatry, Nursing home

## Abstract

**Background:**

Escalation or crisis and the related referral and admission of people with dementia from nursing homes to geriatric psychiatry often results from a complex interplay of disease-related, structural and interprofessional factors. In particular, few contextual links have been identified at the interface between nursing homes and geriatric psychiatry.

**Methods:**

The study followed an exploratory qualitative design. Between May and July 2024, 17 semi-structured interviews were conducted with six nursing home directors/nursing service managers, six nurses, and five general practitioners/specialists. Data were analysed using qualitative content analysis according to Kuckartz and Rädiker. Reporting was based on the COREQ checklist for qualitative studies.

**Results:**

Building on six deductive main categories, we developed 14 subcategories. (1) Multifactoriality and multidimensionality of dementia and behaviour that challenges: 1.1 Characterisation and complexity; 1.2 Impacts. (2) Interventions in behaviour that challenges: 2.1 Trigger identification; 2.2 Non-pharmacological measures; 2.3 Use of psychotropic drugs. (3) Structure-based ability to act: 3.1 Treatment barriers; 3.2 Care conditions. (4) Interprofessional cooperation: 4.1 Information exchange and decision-making; 4.2 Roles and collaboration; 4.3 Transitions. (5) Resources of the setting: 5.1 Professional factors; 5.2 Environmental factors. (6) Coping with the crisis situation: 6.1 Procedures in the crisis development process; 6.2 Individual and psychosocial factors.

**Conclusion:**

Our findings reveal contextual factors in the care of people with dementia and behaviour that challenges that are linked to the escalation of care situations, the development of dementia crises, and to the referral and admission of people with dementia to geriatric psychiatry. These results indicate that structural, personnel and interprofessional changes are necessary at both the personal and professional levels throughout the entire care pathway in order to enable person-centred care for people with dementia. To this end, we recommend the expansion of outpatient geriatric psychiatric care services to improve accessibility and support for nursing staff in crisis situations, clearly defined responsibilities in interprofessional collaboration, dementia-specific training for nursing staff in nursing homes and resilience-building team programmes to improve crisis management are also necessary.

**Trial registration:**

Following positive ethical approval, we prospectively registered the study in the German Clinical Trials Register on 2 May 2024, prior to the inclusion of the first participant (DRKS-ID DRKS00034176).

**Supplementary Information:**

The online version contains supplementary material available at 10.1186/s12912-025-04117-2.

## Introduction

The number of people with dementia is increasing worldwide. In 2019, the estimated number of people with dementia worldwide was 55.2 million. Of all diseases, dementia has become the seventh most common cause of death worldwide, accounting for 3.0% of all deaths in 2019 (more than 1.6 million) [1]. After a diagnosis of dementia, the median survival time is 4.8 years. The median time to admission to a nursing home is 3.3 years but varies depending on the age at diagnosis. Patients who were older when diagnosed with dementia were admitted to nursing homes significantly earlier. For each additional year of life, the time to admission was reduced by an average of 0.3 years (95% CI 0.15 to 0.46) [2]. Advanced cognitive deficits, behaviour that challenges and the associated impairment in activities of daily living are associated with an increased risk of being placed in a nursing home [3]. During the course of dementia, an estimated 90% of those affected develop behaviour that challenges, which cause stress for nursing staff, hospitalisations and inappropriate use of medication [4]. Over 60% of people with dementia living in nursing homes exhibit behaviour that challenges, which complicates nursing care [5]. Behaviour that challenges, especially aggression, cause stress among nursing staff. This worsens the mental and physical health of staff, which can result in burnout, reduced job satisfaction and a deterioration in the quality of care [6]. For people with dementia, behaviour that challenges can lead to difficulties in social interaction with others, as well as to a manifestation of behaviour that challenges due to a lack of communication of needs as a result of increasing language and communication difficulties [7]. Behaviour is not limited to the symptoms of dementia but refers to behaviour as a social construct that is influenced by individual and environmental factors [8]. Behaviour that challenges, specifically aggression, agitation, resistance to care, escape attempts and inappropriate behaviour [9] as well as disinhibition, irritability, restlessness, anxiety, delusions, sleep disorders, hallucinations, deviant motor behaviour and speech disorders [10] are the main reasons why people with dementia are admitted to hospital [9, 10] or especially to geriatric psychiatry [11]. During hospitalisation, patients are exposed to enormous burdens in addition to their acute illness, such as lack of sleep, circadian rhythm disturbances, malnutrition, pain or other complaints. Medication and inactivity due to hospital routine have a negative impact on the recovery phase and influence patients’ vulnerability to new health risks upon discharge. This increased vulnerability is referred to as post-hospital syndrome with the risk of rehospitalisation [12], which is why it is necessary to understand the background surrounding referral processes. Studies indicate that hospitalisations of people with dementia are often avoidable [13–15]. Powell et al. show that nursing home residents diagnosed with dementia were 1.22 times more likely to have an avoidable hospitalisation [15]. Ouslander et al. report that 45% of 100 hospitalisations of nursing home residents were unnecessary [13].

## Background

Some studies show that nursing home residents are admitted to hospital due to changes in their psychiatric or mental state [13], most frequently due to agitation, aggression [16], delirium, as well as confusion and disorientation [17].

There are also potentially avoidable hospital stays that could have been avoided or managed in a nursing home. Temkin-Greener et al. report that in some cases, in addition to somatic diseases such as pneumonia, urinary tract infection, falls and trauma, heart failure and dehydration, psychiatric diagnoses such as psychosis, severe agitation, organic brain syndrome, altered mental status/acute confusion/delirium are also among the diagnostic conditions that lead to potentially avoidable hospitalisations, as they are considered preventable or manageable [18].

Whether admission can be avoided depends not only on the diagnosis or treatment indication, but also on contextual factors. The researchers describe a lack of nursing home resources and psychiatric services, staffing quality, individual resident characteristics (e.g. communication skills), overprescription of antipsychotic medication, etc. as contextual factors that influence the admission of residents with dementia [19]. In addition to behaviour that challenges, social conflicts (e.g. conflicts between staff or relatives and people with dementia) and somatic comorbidities [20, 21] there are other contextual factors associated with the admission of people with dementia. Interprofessional collaboration of healthcare professionals at the interface between geriatric psychiatry and nursing homes is lacking, which is why communication deficits can contribute to an admission [22]. Gaps in the information provided lead to further enquiries, which cause additional work. In addition to inadequate information transfer, there are knowledge deficits regarding the working methods of the others [20]. Furthermore, there are structural and systemic barriers at the interface between geriatric psychiatry and nursing homes [22], such as different routines in the facilities and the lack of adaptability of people with dementia, which can lead to behaviour that challenges [23]. Pre-morbid personality, past trauma and delirium also play a role in the development of behaviour that challenges [24–26]. Guideline-based care, such as one-to-one care and empathetic listening, is often not possible in nursing homes due to limited staff capacity [27]. Pöschel and Spannhorst [20] emphasise that social or care-related stress combined with medical causes can trigger an escalation or crisis, ultimately resulting in people with dementia being admitted to psychiatry. Treatment orders for psychiatry range from optimising medication to treating behaviour that challenges, including sedation, to identifying delirium and diagnosing dementia, to clarifying support and care needs and organising placement in a nursing home for further care [20].

Vroomen et al. [28] define dementia-related crises as ‘*a process where there is a stressor(s) that causes an imbalance requiring an immediate decision which leads to a desired outcome and therefore crisis resolution. If the crisis is not resolved, the cycle continues’.* Poeck et al. [29] make it clear that the initial assessment of acute symptoms (e.g. falls, vital sign abnormalities, conspicuous behaviour) and decisions regarding hospital admissions from nursing homes are made by nursing staff. However, these are strongly influenced by contextual factors such as staff shortages, uncertainty and fear of liability, and limited availability of physicians, which ultimately means that many emergency calls are considered avoidable [29].

When caring for people with dementia in nursing homes who may exhibit behaviour that challenges, this can mean that aggressive and dangerous behaviour, leads to an imbalance or crisis in the nursing home, requiring swift action, such as admission to a geriatric psychiatric ward, in order to resolve the crisis. Pöschel and Spannhorst [20] emphasise that stressors that could still be managed in one support system led to an escalation of the situation and ultimately to decompensation in another context.

The INTERACT (Interventions to Reduce Acute Care Transfers) study [30] shows the extent to which a quality improvement programme can minimise hospital admissions. The programme includes early detection and assessment of acute changes in the condition of nursing home residents, quality improvement tools for analysing the causes of individual transfers, decision support tools for changes in condition that may require a transfer, care pathways for assessing and managing common conditions that can trigger hospital transfers, improved advance care planning, training for nursing home staff and residents, tools for improved communication and documentation between facilities, and tools for medication reconciliation to prevent adverse drug events that can lead to readmission to hospital. The Interact programme reduced hospitalisations among nursing home residents by up to 24% over a six-month period [30].

Hathaway et al. [9] report, that in cases of transfers due to behaviour that challenges, the usual measures for treating medical conditions are often insufficient. Instead, targeted approaches are required that specifically address psychiatric and behavioural changes. These include proactive management directly in the care home, preventive adjustments to the environment, behaviour de-escalation techniques and careful clarification of possible medical causes [9].

The referral of people with dementia from nursing homes to psychiatric clinics is often the result of a complex interplay of contextual factors relating to the complexity of the disease itself, structural and systemic conditions, and interprofessional and patient-centered collaboration between healthcare professionals and relatives [22]. Studies show criteria for avoidable and unavoidable hospital admissions, i.e. admissions that were not preventable [15, 18]. However, the point at which situations escalate, leading to a crisis and thus to a referral or admission, varies from one system to another [20]. Some studies mention referrals of people with dementia to hospitals that focus on somatic treatments or do not mention a psychiatric focus [15, 18].

This study contributes to identifying contextual factors in nursing homes that are associated with the occurrence and management of dementia crises and the referral and admission of people with dementia to geriatric psychiatry. These contextual factors are intended to show when the breaking point is reached, i.e. under which circumstances an admission is necessary. In this context, the perspective of healthcare professionals is crucial in order to obtain indications for measures that could address the needs. The perspective of healthcare professionals at the interface between geriatric psychiatry and nursing homes remains largely under-researched, particularly with regard to the development of dementia crises and the conditions that influence referrals or admissions. Due to the special nature of the topic and its strong dependence on the system, particularly in the German healthcare system with its specific admission processes, this area has been little researched to date. The contextual factors identified in this study can serve as a basis for investigating comparable aspects in other healthcare systems. In this way, the results contribute to a more targeted approach to international research on avoidable hospital admissions.

## Methods

This study is based on an integrative review [22], which identified contextual factors for the (re)admission to geriatric psychiatry of people with dementia and behaviour that challenges from nursing homes. It was found that crisis situations often lead to the admission of people with dementia and that the contextual factors associated with admission to geriatric psychiatric wards are ‘*crisis-determining factors*’ [22].

### Aim

This study aims to identify the contextual factors from the perspective of healthcare professionals at the interface between geriatric psychiatry and nursing homes. This should contribute to an improved understanding of crisis situations, challenges and gaps in relation to (re)referral and (re)admission processes and should help us to formulate initial assumptions about how certain chains of factors could contribute to the emergence of crisis situations and the decision to refer people with dementia to geriatric psychiatry. These findings should contribute to the long-term development of a needs-based interface intervention to be established in a specific context. The aim is to bring about structural, organisational and personnel changes at both the personal and professional levels throughout the entire care pathway in the future, in order to better avoid unnecessary psychiatric hospital admissions of people with dementia in crisis situations.

The following research question was investigated:

How do healthcare professionals describe the contextual factors related to the (re)referral and (re)admission of people with dementia and behaviour that challenges from nursing homes to geriatric psychiatry?

## Study design

### Theoretical framework

An exploratory qualitative study design was pursued to explore and describe the existing under-researched topic [31]. The results were analysed using the qualitative content analysis according to Kuckartz and Rädiker [32]. Semi-structured interviews were conducted between May 2024 and July 2024 with nurses, nursing home directors/nursing service managers and general practitioners (GPs)/specialists at the interface between geriatric psychiatry and nursing homes in rural areas in Germany. The study received a positive ethics approval (application number 24 − 008) from the responsible ethics committee, the German Society for Nursing Science (Deutsche Gesellschaft für Pflegewissenschaft, DGP). The study was then prospectively registered in the German Clinical Trials Register (Deutsches Register Klinischer Studien, DRKS, https://www.drks.de/search/de) on 2 May 2024, prior to the inclusion of the first participant (DRKS-ID DRKS00034176).

### Data collection

We conducted a qualitative study using a semi-structured interview guide and a questionnaire that collected socio-demographic data (age, gender, profession, additional qualifications, years of professional experience and scope of employment). The interviews were problem-oriented and followed Witzel’s problem-centred interview approach [33]. The problem-centred interview (PCI) is an open, semi-structured interview method that allows the interviewee to talk freely but always returns to a specific problem [34]. We derived the interview guide based on key topics from the current state of research. This was based on the preliminary results of the integrative review conducted previously [22]. We used the subthemes and categories identified there as the basis for developing the individual sections and interview questions in the interview guide. The interview guide can be found in Table S1 in the supplementary material. The respective subsections of the interview guide contain guiding questions or prompts for narration, key points that offer the possibility of asking questions if something is not mentioned, questions about maintenance and specific questions based on the SPSS approach: Collecting (German: ***S****ammeln*), Examining (German: ***P****rüfen*), Sorting (German: ***S****ortieren*), and Subsuming (German: ***S****ubsumieren*) [35]. Before data collection began, a pilot interview was conducted with an experienced nursing professional from the fields of psychiatry and long-term care. The aim was to check the guide for comprehensibility, clarity, appropriateness of wording and duration. The complete interview confirmed the suitability of the questions for the target group (physicians, nursing staff, nursing managers), so that only minor linguistic adjustments were necessary. The interview questions were not provided to participants in advance in order to encourage spontaneous responses.

The interviews were conducted face-to-face in the participants‘ working environment, i.e. in medical practices and in nursing homes. It was ensured that only the interviewer and the interviewee were present during the interviews, no third person. There were no subsequent refusals to participate or interruptions to interviews. The individual interviews were audio recorded, and field notes were made to situational or non-verbal expressions. The interviews were completed in full before the systematic analysis began.

### Participants and settings

The study was conducted in rural areas in Germany. Nursing home directors/nursing service managers, nurses, GPs and specialists (in-patient and out-patient) were selected as relevant healthcare professionals for the qualitative interviews. Based on their professional experience, they have in-depth insights into the referral process of people with dementia and behaviour that challenges to geriatric psychiatry.

Those who refer patients to geriatric psychiatry are primarily specialists (psychiatrists, neurologists) and GPs [20], as well as the psychiatric outpatient clinic (Psychiatrische Institutsambulanz, PIA) [11]. The psychiatric outpatient clinic is a form of outpatient treatment affiliated with a hospital that cares for patients with severe, chronic or long-term mental illness in order to avoid or shorten hospital stays [36]. Furthermore, nurses in nursing homes are often responsible for the referral of residents to hospital and sometimes carry out the referral without consulting specialists or GPs [37]. Residents are often cared for by the PIA due to multiple mental disorders [38]. Psychiatrists from the PIA were interviewed to determine the extent to which the PIA is involved in the decision-making process regarding referral and admission to geriatric psychiatry.

Furthermore, the management level in nursing homes has an influence on the workload of nurses [39] on the implementation of person-centred care and on the psychosocial climate [40]. Nursing home directors/nursing service managers were included in the interviews to draw conclusions about the extent to which they are involved in the process of referring residents to geriatric psychiatry.

### Participant selection

In the sense of targeted (selective) sampling, a preliminary determination of the interview participants took place based on an internal data transfer from the documentation system of a geriatric psychiatry. This made it possible to determine the population of all nursing homes and GPs and specialists who referred patients to geriatric psychiatry in 2022 and 2023 (selection of two wards that primarily treat people with dementia and behaviour that challenges).

From the population (all nursing homes and GPs/specialists who referred patients with dementia to geriatric psychiatry in 2022 and 2023), a targeted sample was used to select those nursing homes and GPs/specialists who referred patients with dementia to geriatric psychiatry due to behaviour that challenges. 56 nursing homes and 99 GPs and specialists were identified. The nursing homes, GPs and specialists who had most frequently referred people with dementia were asked for an interview. In the end, 36 GPs and specialists and 29 nursing homes were contacted. Our goal was to interview five nursing homes (five nurses and five managers) and five physicians (GPs/specialists). However, during the recruitment process, another nursing home agreed to participate, so that ultimately six nursing homes (six nursing home directors/nursing service managers and six nurses), three GPs and two specialists (from the PIA) were interviewed.

The inclusion criteria were professional experience in the context of referral or re-referral of people with dementia, legal majority and sufficient language skills to answer the interview questions in German.

### Data analysis

The interviews were professionally transcribed according to the transcription guidelines of Dresing and Pehl. Their semantic content rules include literal transcription, writing down short answers (hmm, yes, exactly/right), recording significant pauses, noting emotions and intonation, and standardising the design of the transcripts [41]. The following analysis of the interviews was based on Kuckartz and Rädiker’s qualitative content analysis (*preparation of the material, development of deductive categories, coding of the material with deductive categories, inductive expansion of the categories, revision and refinement of the category system, systematic coding of the entire material and evaluation, analysis and interpretation*) [42]. In the step ‘*Preparation of the material*’, we imported the transcribed interviews into the MAXQDA 2024 software and anonymised all names of people and/or places. In the next step, *‘Development of deductive categories*’, we defined main categories based on deductive predefined themes, which were then coded during the analysis in the step *‘Coding the material with deductive categories’.* In the step *‘Inductive expansion of categories’*, we developed inductive subcategories from the data material and further differentiated them. First, the main author coded and categorised all data using MAXQDA 2024 software. Then, we (main author and co-author) discussed and, where necessary, revised the category names and adjusted the hierarchical structure (step: ‘*Revision and refinement of the category system’*). In the step ‘*Systematic coding of all material and evaluation’*, we systematically reviewed each individual code together and adjusted it if necessary. In the next step, *‘Analysis and interpretation’*, we worked out the results from the previously coded data, described them systematically and substantiated them with appropriate quotations from the material. In order to make the research results of the qualitative interviews in German accessible to a global audience, we translated the quotes using DeepL Pro and had them reviewed by a native speaker. All interpretations were discussed between two researchers to ensure consistency. When forming the categories, care was taken to use more general terms that reflect the multifaceted nature of the content without narrowing the data too much. This allowed both dominant topics and less frequent but relevant aspects to be represented.

Already during the data collection phase, the content field notes taken during the interviews revealed an increasing thematic consolidation: key content was repeated, and new interviews only led to minor additions to the content. This picture was confirmed in the subsequent qualitative content analysis, in which no new relevant categories or subcategories were identified during the coding of the last four interviews. Instead, existing thematic structures were confirmed. We therefore assume that content saturation in the sense of qualitative research has been achieved.

We validated the data communicatively as part of a member check. The purpose of this communicative validation or member check was to determine the extent to which the healthcare professionals interviewed agreed with, rejected or wished to supplement the data obtained from the interviews. Five interviewees took part in the communicative validation, including three nursing home directors and two nursing professionals. In a face-to-face meeting, we presented the results of the interviews, i.e. the main and subcategories with selected quotes (and interview codes, line numbers in the transcript), to the plenary session. General questions regarding comprehension could be asked after each section. The following questions were then discussed in plenary based on McKim’s structured approach [43]:

Having heard the results, what are your general thoughts? How accurately do you feel the results reflect your experiences? What could be added to the results to better capture your experiences?

We first documented the answers to each question in writing in the form of notes and made them visible to all participants in the plenary session. We then asked the participants to check whether these notes accurately reflected their statements. If the written version did not fully capture the meaning, they were able to make corrections or additions. This ensured that the documented data corresponded to the participants’ intentions. The feedback process served solely for the validation of the presentation; there was no influence on the content or opinions.

## Research team and reflexivity

### Personal characteristics

The interviews were conducted by the two female authors. The first author (IHR) holds a Master of Science in Advanced Practice Nursing and has many years of professional experience both as a nurse and in the role of an advanced practice nurse. At the time of the study, she worked as an advanced practice nurse, also worked as a research associate specialising in qualitative data analysis, and was in the process of obtaining a doctorate in public health. She is already familiar with semi-structures interview methods, explicitly Witzel’s problem-centred interview method from her nursing science studies and also has experience with the method from a previous thesis. The co-author (VFU) holds a doctorate in nursing science and has professional experience as a nurse, as an advanced practice nurse, and as a lecturer at a university of applied sciences. She has extensive knowledge and experience of qualitative interview methods from her many years of study and teaching. She also used qualitative guided interviews in her own doctoral thesis.

Almost all interviews (*n* = 16) were conducted by the first author (IHR) to ensure consistency. To minimise potential response bias, one interview was conducted by the co-author (VFU) because the first author and the person interviewed had previously worked together professionally. This ensured that the interviewers had no personal or professional relationship with the participants in the respective interviews. As the first author already had experience in conducting problem-centred interviews, the co-author was informed in advance about the methodological specifics of the study and provided with relevant literature.

### Relationship with participants

No relationship was established with the participants prior to the start of the study. The participants were informed about the personal goals (obtaining a doctorate from IHR) and reasons for conducting the research (identifying problems to improve interface management between geriatric psychiatry and nursing homes). The participants were aware of the function and institutional affiliation of the researchers, so it cannot be ruled out that deficits were formulated in a more reserved or positive manner.

## Results

The study results were reported in accordance with the Consolidated Criteria for Reporting Qualitative Research (COREQ) [44].

A total of 17 participants aged between 20 and 69 were interviewed in person. The duration of the interviews varied between 28 and 88 min. The participants’ professional experience ranged from one to over 30 years, their level of employment was between 30% and 100%. Some participants had specialist medical or nursing qualifications. On the medical side, the specialist titles psychiatry and psychotherapy, general medicine and neurology were mentioned. In addition, several additional qualifications were named, including palliative medicine, geriatrics, and diabetology. In nursing, state-recognised further training in oncology, geriatric psychiatry, case management (German Society for Care and Case Management) and quality management were reported. Medical participants also mentioned qualifications in quality management (see Table 1). The intention to participate among GPs, specialists and nursing homes with high referral rates was low. The reasons for the rejection were a currently vacant management position or a general lack of interest in surveys and interviews. This means that healthcare professionals who referred patients less frequently were interviewed.

**Table 1:** Sociodemographic data of the participants

Age in years	*n*	Gender	*n*
20–29	1	Female	15
30–39	2	Male	2
40–49	3	Diverse	-
50–59	8		
60–69	3		
**Profession**	**n**	**Additional qualifications**	**n**
Nursing home director	3	Oncology	1
Nursing service manager	3	Case management	1
Nurse	6	Quality management	4
General practitioner	3	Geriatrics	2
Specialist in psychiatry	2	Psychiatry/ geriatric psychiatry	4
		Diabetology	2
		Neurology	2
		Palliative medicine	3
		Internal medicine	2
		General medicine	1
**Years of professional experience**	**n**	**Employment in %**	**n**
01–05	3	100	12
06–10	2	80	1
11–15	1	75	1
16–20	4	60	1
21–30	5	50	1
More than 30	2	30	1

### Contextual factors associated with dementia crises and the related referral and admission processes

The following results present the contextual factors associated with dementia crises and the related (re-)referral and (re-)admission of people with dementia and behaviour that challenges from nursing homes to geriatric psychiatry from the perspective of the healthcare professionals. Fourteen subcategories were identified from the six deductive categories through qualitative content analysis. We identified the following categories and subcategories: (1) Multifactoriality and multidimensionality of dementia and behaviour that challenges: 1.1 Characterisation and complexity; 1.2 Impacts. (2) Interventions in behaviour that challenges: 2.1 Trigger identification; 2.2 Non-pharmacological measures; 2.3 Use of psychotropic drugs. (3) Structure-based ability to act: 3.1 Treatment barriers; 3.2 Care conditions. (4) Interprofessional cooperation: 4.1 Information exchange and decision-making; 4.2 Roles and collaboration; 4.3 Transitions. (5) Resources of the setting: 5.1 Professional factors; 5.2 Environmental factors. (6) Coping with the crisis situation: 6.1 Procedures in the crisis development process; 6.2 Individual and psychosocial factors.

**Fig. 1:**
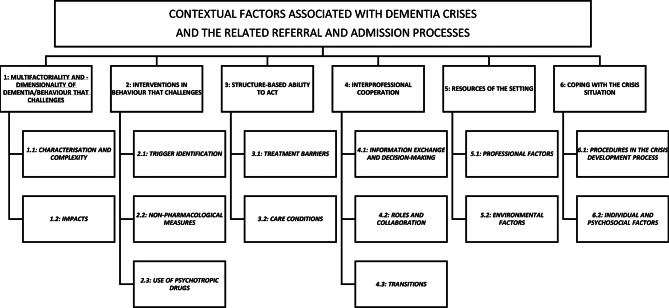
Overview of the main categories and subcategories

The hierarchically structured presentation of the main and subcategories can be seen in Fig. 1. The numbering of the main categories does not imply that category one is more important than category six. Rather, it illustrates that all contextual factors play an essential role in the referral and admission processes and can vary in importance depending on the specific setting.

A more detailed explanation of the content of the main and subcategories, including key quotations and coding frequencies can be found in the analysis grid in Table S2 in the supplementary material. The coding frequencies in the analysis grid are only meant to make the category summaries in the analysis easier to follow and show which topics the respondents brought up a lot in their everyday work and are therefore potentially relevant in practice. It’s not meant to be a quantitative interpretation in terms of statistical frequencies.

The contextual factors associated with dementia crises that are related to the referral and admission of people with dementia to geriatric psychiatry are explained below:

The main category **‘MULTIFACTORIALITY AND MULTIDIMENSIONALITY OF DEMENTIA AND BEHAVIOUR THAT CHALLENGES’** reflect the various factors and dimensions associated with dementia and the resulting behaviour that challenges that ultimately lead to crisis situations in the care environment and to referral and admission to geriatric psychiatry. The two subcategories ‘characterisation and complexity’ and ‘impacts’ could be identified.

The subcategory **‘CHARACTERISATION AND COMPLEXITY’** shows that the severity of dementia and the type and intensity of behaviour that challenges lead to escalation situations in nursing homes and influence admission to psychiatry. Especially aggression towards oneself or others, agitation and restlessness, shouting and screaming is difficult to manage and leads to referral: *‘What is always very difficult for us is aggressive behaviour. When a resident is auto-aggressive but is also aggressive towards others’* (B7, pos. 5, nursing home director). Refusal behaviour is also problematic, especially if other somatic diseases or psychiatric disorders exist which need regular medications: *‘But of course with dementia there is also refusal to eat, refusal of food, refusal of fluids. If the patients then get into a state where they are no longer supplied. In fact, we have already had patients who refused to be washed, who then somehow, who simply no longer allowed themselves to be cared for, where we then also said, maybe it’s time for geriatric psychiatry’* (B15, pos. 16, GP). The severity of behaviour that challenges plays an elementary role in escalating situations and the related referrals: *‘Sometimes you reach your limits, depending on the severity of the challenging behaviour, right? Sometimes you’re helpless and then you don’t know yourself’* (B14, pos. 3, nurse).

The subcategory **‘IMPACTS’** goes beyond the intensity of the behaviour that challenges to illustrate the serious effects that the behaviour can have on the person with dementia and others. The interviewees emphasised the danger to themselves and others, the suffering and helplessness of people with dementia due to their condition and the high level of care required: *‘Yes, if it simply becomes too abusive or if it becomes too impulsive, then it is also a danger to the employees, yes. So, we also have to protect these employees. Or other residents as well’* (B9, pos. 15, nursing home director). They also mentioned the stress levels for staff and other residents, as well as the behavioural contagion: *‘The staff also become more restless. They then work completely differently. They’re somehow so agitated internally, so agitated. And of course that is then transferred to the other residents’* (B4, pos. 7, nursing service manager).

The main category **‘INTERVENTIONS IN BEHAVIOUR THAT CHALLENGES’** shows the measures used to prevent and treat behaviour that challenges in dementia. The interventions are closely associated with admission, as unaddressed causes of the behaviour and inadequately conducted or ineffective measures can lead to the worsening of the behaviour and situations escalating into referral. The three subcategories ‘trigger identification’, ‘non-pharmacological measures’ and ‘use of psychotropic drugs’ were found.

The subcategory ‘***TRIGGER IDENTIFICATION*’** includes a lack of awareness and insufficient reflection on the behaviour and situation of people with dementia. The behaviour and situation analysis are not in-depth enough and a quick solution is often sought, which is why referral to geriatric psychiatry is often seen as a quick fix: *‘And then I always say: What has just triggered him? Because these are always moments when they get triggered. Did you go too fast? Did you not take enough time?’* (B11, pos. 187, nurse). Nursing staff often focus too little on the life, biography and identity of the person with dementia and biographical findings are not re-evaluated enough in the current context. Physicians often do not sufficiently clarify somatic causes, especially pain: *‘And we have to fight hard to ensure that a somatic assessment is carried out first and that not everything is attributed to the mental illness in the first place. And it’s urinary tract infections, pain and so on that tend to be forgotten, and then we have to take more medication or even more without considering the somatic component’* (B10, pos. 49, psychiatrist).

The subcategory ‘***NON-PHARMACOLOGICAL MEASURES‘*** involves the importance of responding to physical and psychological needs and relationship work: *‘And just being with him sometimes. Sometimes all it takes is this appreciation, attention, a caress, a hug. Everything that a person actually needs. These needs. Sometimes I don’t even have to tell them but just give them a hug. This feeling: Oh God, no, I’m being taken seriously, I’m being seen. I feel someone. […] And you can actually achieve a lot with that, can’t you?’* (B14, pos. 35, nurse). Lack of attention to people with dementia, missing one-to-one care, poor daily structure and organisation and, above all, failure to eliminate triggers can lead to behaviour that challenges and escalating situations and referral to psychiatry: *‘I have a resident situation in my head where a resident had reacted very aggressively to women, but totally/ you could get him down again when someone came in a uniform. We realised this because the staff had to call the police twice because he was so aggressive and was throwing all the furniture. And the moment a uniformed person stood in front of him, there was silence. So that’s a familiar story. Or that it’s the gender that triggers it. If someone reacts badly to a man, we try to only send women into care, and vice versa, yes’* (B7, pos. 27, nursing home director). The interviewees also highlighted the relevance of respectful, person-centred communication.

The subcategory ‘***USE OF PSYCHOTROPIC DRUGS****’* includes the unreflected and untargeted use of psychotropic drugs and the lack of evaluation to discontinue medication, which can lead to further consequences and associated admissions or readmissions to psychiatric wards. According to the respondents a specific risk-benefit assessment is carried out before administration: *‘And you don’t always have to go straight to the on-demand medication to sedate the resident. I first have to find out the reason why the resident has displayed the challenging behaviour right now, right now. And often we can actually validate the resident’s behaviour and pull them out and go for a walk rather than administering half a Tavor or something like that’* (B12, pos. 31, nursing service manager). The administration of medication, e.g. oral or depot preparations, is often difficult in nursing homes and the medication therefore only has a limited effect: *‘Well, I’ll say, in our environment it’s difficult to administer them because we can usually only give them orally’* (B6, pos. 51, nurse). Psychotropic medication is sometimes used for escalation prevention and that the usage is dependent on nursing staff. Psychotropic drugs are also used when all other measures have been exhausted and have proven to be insufficiently effective or when the behaviour is no longer tolerable: *‘But that is hardly tolerable. There are also visitors, and if they are now defecating in the corridor or are really so aggressive, what should be done? Of course, he was also on a high dose of risperidone. So, I was really at the end of my tether’* (B10, pos. 31, psychiatrist). Some interviewees reported that psychotropic drugs sometimes have paradoxical effects. Sedative drugs are also used to manage daily care in order to increase the residents’ ability to cooperate. They also stated that somatic and psychiatric side effects are accepted to avoid disturbing the environment or to reduce the suffering of people with dementia. *‘It’s necessary, there’s no other way. But we are aware of all the advantages and disadvantages of administering medication in geriatrics’* (B1, pos. 75, GP).

The main category ‘**STRUCTURE-BASED ABILITY TO ACT’** encompasses the subcategories ‘treatment barriers’ and ‘care conditions’.

The subcategory ***‘TREATMENT BARRIERS’*** refers to factors and external circumstances that hinder the treatment of people with dementia. It emphasises that there are limited treatment options and uncertainty in dealing with behaviour that challenges in dementia, which leads to repeated crisis situations and readmissions. In geriatric psychiatry, too, options are limited, so people with dementia are discharged again on the grounds that no further treatment is possible, including effective non-pharmacological treatment methods: *‘So we still have this case at the moment. It will stay forever, that won’t change. But this constant screaming is a burden on the residents. She’s no longer admitted because she’s completely out of therapy’* (B16, pos. 39, nurse). Furthermore, diagnostic and therapeutic barriers exist due to limited specialist medical care and generally limited availability of physicians, as well as a lack of dementia specialisation in nursing homes. A lack of technical equipment, e.g. electrocardiogram machine, can lead to the interruption of therapeutic measures: *‘Sometimes we don’t continue things because, for example, we can’t write an* electrocardiogram *for a patient in a nursing home.’* (B15, pos. 99, GP). Regulatory hurdles make it even harder to care for people with dementia in nursing homes, for example, because nursing staff aren’t allowed to refer patients or prescribe medication, nothing can happen without a physician’s order, and there are lots of steps to go through before someone can actually be admitted: *‘So the main problem […] is this admission itself, because you have to take various steps first. That means I have to inform the public order office [German: Ordnungsamt] first. Then, if I’m lucky, they’ll come early. Then I first have to explain: What’s going on anyway? They then decide whether to make a referral or not? Or the police have to be called after all. That delays everything.’* (B14, pos. 47, nurse). COVID-19 further exacerbated treatment barriers of people with dementia due to the requirements imposed by the health authorities, such as the mandatory wearing of masks for residents. This led to a deterioration in dementia and behaviour that challenges due to contact restrictions and isolation measures. Admission to geriatric psychiatry was made even more difficult by requirements such as negative tests, and in some cases admission was categorically refused by the psychiatric department.

The subcategory ***‘CARE CONDITIONS’*** refers to the organisational and practical working conditions. It highlights the difficulty of getting someone admitted to the clinic, e.g. patients are only admitted in an emergency, and elective admissions are almost impossible, and the clinic often questions the necessity of admission. There is also reduced bed capacity due to lack of staff in the geriatric psychiatry and therefore long waiting times: *‘Most of the time we have the problem, and it’s also clearly due to a lack of staff or capacity, that there’s no space available in the [name clinic]. That’s also a problem’* (B14, pos. 47, nurse). Staff shortage and time deficits exist in all areas, e.g. medical practices, nursing homes and in the geriatric psychiatry, the entire healthcare system is overloaded. Therefore, there is an inadequate diagnostic (GP/specialist) and a poor patient care (nursing homes): *‘At night, in the nursing homes where I’ve worked so far, there’s a registered geriatric nurse with […] almost 45 patients and when you imagine that she starts washing at the front, there are patients with dementia, there are patients who can’t go to the toilet themselves. And then she starts at the front and finishes at the back. And if one of the patients then somehow gets in the way, that’s not possible in a nursing home. There are no staff resources to deal with that’* (B15, pos. 8, GP). This is also a reason for the emergence of behaviour that challenges and escalating situations: *‘And of course you have to be honest and say that some of the challenging behaviour is of course also the result of staff shortages’* (B10, pos. 75, psychiatrist). Interviewees also emphasised that the conditions under which care must be provided result in people with dementia receiving inadequate care and ultimately being referred to psychiatry.

The main category ‘**INTERPROFESSIONAL COOPERATION’** highlights the importance of healthcare professionals at the interface between geriatric psychiatry and nursing homes and their interaction in the context of the referral and admission process. Three subcategories were identified: ‘information exchange and decision-making’, ‘roles and collaboration’ and ‘transitions’.

The subcategory **‘INFORMATION EXCHANGE AND DECISION-MAKING’** is characterised by insufficient information transfer, missing or poorly comprehensible information in transfer forms and in the discharge letter from the geriatric psychiatry. In some cases, no transfer forms are provided upon discharge, or the discharge letter is forwarded to the GP and the nursing home but not to the PIA. The interviewees reported on a lack of transparency regarding the background to the diagnosis, changes in medication and the implementation of psychosocial measures, which result in a higher workload due to queries: *‘Well, what I experienced again and again is that information was only sparsely available. That […] the triggering situations were not really described or communicated. Yes. That is the main problem, […] that it must be researched’* (B2, pos. 60, psychiatrist).

Furthermore, the interviewees stated that communication between the nursing home and the geriatric psychiatry department takes place at such short notice, i.e. only shortly before discharge. In addition, communication between GPs and pharmacies is inadequate, with several medication plans in place, meaning that nursing staff do not know which plan to follow. Specifically, communication between nursing homes, between PIA and PIA, and between geriatric psychiatry and PIA is inadequate, and there is a lack of communication between professional groups and institutions when it comes to changing medications. In addition, some respondents emphasised the unreliability of information and reported that information is often embellished and data is outdated. They also reported that relatives sometimes conceal dementia diagnoses and behaviour that challenges, and that information from somatic hospitals and geriatric psychiatric wards is withheld in some cases: *‘Sometimes these inaccurate statements from geriatric psychiatry. […] I experienced a situation where a care nurse really wanted to refer someone to us just because she liked them and felt sorry for them, but we said we couldn’t take the person in because she had embellished it first. And only after we started to ask the right questions did we notice her behaviour: Oh, she wants to sugarcoat the fact that we’re taking him and that won’t work. So, we really need honesty here, because that’s the prerequisite for dementia, because we can’t look inside the resident’* (B14, pos. 78, nurse). The decision-making process is also important to the respondents, in addition to the pure exchange of information. They stated that hospital admission and the competence of other professional groups are questioned by geriatric psychiatry and that there are differing opinions as to when admission is necessary. These differences of opinion exist primarily among the medical profession; some interviewees mentioned that some geriatric psychiatrists often do not trust the judgement of GPs regarding the necessity of admission, nor do they trust that of outpatient specialists. An additional challenge arises when patients do not exhibit behaviour that challenges in a clinical setting, or do not exhibit it to the same extent. Furthermore, there are also differing opinions among nursing staff in nursing homes about the necessity of admission.

The subcategory **‘ROLES AND COLLABORATION’** includes the inadequate availability and long waiting times of the PIA and the GP. Some participants pointed out that nursing staff have to chase GPs for referrals and that availability is poor, particularly at weekends, and that the on-call physician is often ‘unsuitable’ due to a lack of expertise in dementia. They also reported that it is difficult for the GP to reach colleagues in the clinic, that the GP only visits nursing homes very rarely for financial reasons and that the general condition of the residents has often deteriorated by the time the GP arrives. According to the interviewees, discharge from geriatric psychiatry and first admission to a nursing home only take place on days when the GP is available: *‘When he [the resident] comes for the first time, we also make sure that we don’t do this admission on certain days, i.e. not on Wednesdays or Fridays, because we always have to make sure that the physicians are available when, let’s say, the familiarisation period lasts a little longer. That’s another important point’* (B12, pos. 55, nursing service manager). The distribution of roles and hierarchies are a key issue for the interviewees. They reported that unclear roles and hierarchies hinder patient care. There is often disagreement among physicians regarding medication, which leads to the GP discontinuing the medication prescribed by the specialist. The responsibilities between physicians are often unclear, especially in the case of dementia. The allocation of roles and hierarchies are also made clear by the fact that the GP is solely responsible for determining the indication and issuing referrals and not the nursing staff. In addition, some interviewees mentioned, that geriatric nurses are sometimes disparaged with regard to their competence: *‘Competence dispute, GP says he’s better than the specialist: “We can sort it out here too.” The specialist in turn says: “That’s no longer our job, please let the GP sort it out.” The nurse says: “You both have no idea. I’m here 24/7, you haven’t got a clue, why don’t you listen to me?” So, it’s a three-way relationship’* (B13, pos. 83, nursing service manager). Another aspect is the lack of knowledge of each other’s working methods, the different professional groups and settings do not know how the others work. The interviewees also highlighted that there is no interprofessional perspective, there is a discrepancy between psychiatric and somatic treatment, and there is a time lag in the perception of the behaviour that challenges by those involved, as GPs do not disturb restless patients at night because they are not present.

The subcategory ‘**TRANSITIONS’** includes transfers from nursing homes to geriatric psychiatric wards and discharge from geriatric psychiatric wards to nursing homes. Regarding the transition geriatric psychiatry, outpatient services, especially PIA, are perceived as a relief and a quicker solution. PIA is indicated as the first point of contact for behaviour that challenge for referral to geriatric psychiatry. The lack of involvement of the PIA is perceived as a burden. The interviewees stated that they consult the GP at an early stage for referral to the PIA to be able to intervene early and avoid crises. According to the respondents the PIA has often limited resources and neurological care in nursing homes is inadequate: *‘We then took in a woman with severe dementia here. She was only ever treated by her GP. At some point, it just didn’t work anymore. And the GP issued referrals. It took us nine months until the PIA was here. […] And if we can’t bridge that […] with a GP, then we have a problem here’* (B17, pos. 111, nursing service manager). Regarding the transition from geriatric psychiatry to nursing homes, some interviewees reported that discharge is often not announced in good time and that too few medications are provided. In addition, discharge to a nursing home happens more quickly because follow-up care is guaranteed there: *‘You also have to admit that there are also many so-called “bloody” discharges […] It’s not the case that people, if they’re good, are often still there for a long time, but then it’s more or less stopped and then […] they’re put back into the nursing home, because you know they’re well looked after.’* (B10, pos. 45, psychiatrist). An on-site assessment of patients in psychiatric wards prior to admission to a nursing home and a trial discharge to a nursing home are considered helpful. Furthermore, the participants desire for aftercare provided by PIA or specialists: ‘*I miss the aftercare, that you always have someone on site or that some-one comes round after two or three days or after a week and says: “And how is it with the resident? Is it going well? Is everything all right?”’* (B14, pos. 53, nurse).

The main category **‘RESOURCES OF THE SETTING’** includes the professional and environmental resources associated with the onset of dementia crises and admission to geriatric psychiatry. This is not just a question of the lack of resources in terms of quantity, but rather the quality of the resources available or the quality of care. The two subcategories ‘professional factors’ and ‘environmental factors’ were identified.

The subcategory **‘PROFESSIONAL FACTORS’** shows that a lack of language skills and different understanding of care among international staff and a lack of knowledge and skills often lead to behaviour that challenges from people with dementia: ‘*We have more and more nursing staff […] with a migration background. Language is often a barrier. Employees with a migrant background bring their culture and their understanding of the profession with them. They do not always fit in with that, so they are not always congruent’* (B17, pos. 9, nursing service manager). Some interviewees emphasised that limited specialist knowledge and little practical experience lead to crisis situations and to referrals to geriatric psychiatry, where nursing staff are regarded as specialists. One interviewee also noted that training, e.g. in psychiatric nursing, can be a barrier and lead to ‘over-treatment’ of people with dementia, as their behaviour is sometimes over-interpreted and seen as a symptom of the disease. A lack of reflection on escalating care situations also leads to referrals, as these are not considered individually but follow a standard approach: *‘I would say that this has happened due to employees who were new to our nursing home. Who simply experienced things differently and acted differently before-hand. Who didn’t intervene and evaluate in advance, who were simply on day shift. And they couldn’t cope with it’* (B13, pos. 121, nursing service manager).

In addition, the participants described a lack of practical experience among new employees in nursing homes. They perceive professional experience as a resource in nursing homes for maintaining the ability to act in difficult situations, with experienced staff considered a preventive measure against hospital admissions: *‘So I would say that there is an absolute correlation between the lower the level of expertise, the quicker […] the desire to refer patients, of course.’* (B10, pos. 65, psychiatrist). They also pointed out that not being able to build relationships because of frequent staff changes makes it harder to provide good care and that continuity and relationships have a positive effect on how residents behave. They mentioned that a bad relationship between a nurse and a resident in a nursing home often means that the resident keeps behaving the same way after a stay in a geriatric psychiatric ward. The interviewees emphasised that care assistants and volunteers are seen as a great relief, as they are responsible for daily activities and therapeutic measures that nursing staff are unable to perform due to other demands. Attitudes towards relatives are somewhat ambivalent. On the one hand, they are perceived as a resource and a relief in the professional process, but also as a trigger or a burden. Relatives can make the referral process for people with dementia to geriatric psychiatry easier by contacting their GP and requesting a referral. The burden becomes clear when relatives delay or prevent admission because they have concerns and false assumptions about the procedures in psychiatry. It is also a burden when palliative decisions are made more difficult due to a lack of professional knowledge on the part of relatives.

The subcategory ‘***ENVIRONMENTAL FACTORS*’** includes structural barriers such as room size, lighting and odours, which can affect the well-being of people with dementia. The participants reported that the atmosphere of the ward/residential unit is important, as it provides a sense of security and a feeling of being at home. They also mentioned that in some cases there are no places where people with dementia can retreat which can lead to crisis situations. There are more and more stimuli that cause sensory overload, affecting both staff and residents. Quick care procedures due to staff shortages, a hectic pace and disregard for the routines of people with dementia lead to behaviour that challenges. Individual care is often not possible due to rigid shift schedules and daily routines. Routines in nursing homes, such as rules regarding order and cleanliness, cause stress among residents. The participants reported that staff resources are needed to create a daily structure: ‘*If I have enough resources, i.e. enough staff, to create a daily structure to make the patient somehow tired during the day. Or if they somehow don’t understand or misunderstand something, to have a calming effect on them. If I have the resources for that, then the situations don’t escalate’* (B15, pos. 125, GP). According to the interviewees, a common reason for recurring escalating situations and readmissions to geriatric psychiatry is when wandering cannot be controlled and the nursing home or unprotected residential unit is too unsafe for these residents. Residents with a tendency to wander off are often sedated with medication to prevent them from leaving. Alternatively, a referral to geriatric psychiatry may be arranged if an adjustment to the living situation is necessary. This is particularly the case if the previous care home does not have a secure living area and is therefore not legally authorised to prevent residents from leaving the facility: *‘We only have protected units, we don’t have closed units. This means that we can’t keep someone here who really tends to run away, who really wants to leave every minute, every two minutes. […] And then we can’t keep someone who is a danger to him or herself or to others, or lock rooms, for example. Five-point fixation, three-point, it doesn’t matter. We can’t do that here’* (B13, pos. 164, nursing service manager). Another contextual factor leading to dementia crises and to referral is a lack of dementia specialisation, i.e. a lack of a dementia concept or a separate dementia specialised unit. Sometimes residents with different psychiatric disorders are mixed together in the residential units, which can lead to uncertainty and conflicts among residents who are still orientated towards time, place and/or person.

Nevertheless, interviewees emphasised that hospital stays are disastrous for people with dementia due to the change in environment and the resulting consequences, such as deterioration in cognitive functions and activities of daily living. They reported that the condition of patients sometimes deteriorates during inpatient treatment and that residents need time to adapt to the new environment (acclimatisation phase) even after treatment in geriatric psychiatry, otherwise new escalating situations and readmissions arise. They also pointed out that different environments lead to different behaviour. The changed environment in geriatric psychiatry often leads to the disappearance of behaviours that reappear after discharge to the nursing home. Problems that did not occur in geriatric psychiatry persist in the nursing home despite successful psychiatric treatment: *‘The circumstances in the clinic differ from those in everyday life in a nursing home. And sometimes something that was perceived as a positive development in a clinic […] was seen as an improvement that did not remain as constant in everyday life, but instead things that had previously been problematic reappeared, where there was supposedly no improvement at all’* (B2, pos. 62, psychiatrist).

The main category **‘COPING WITH THE CRISIS SITUATION’** addresses the handling of crisis situations, the associated influencing factors and the related referral decisions. It is not just a question of a lack of structural conditions, or insufficient resources but above all of their impact on the psychological and individual level of healthcare professionals and institutions and their breaking point. The subcategories ‘procedures in the crisis development process’ and ‘individual and psychosocial factors’ were identified.

The subcategory **‘PROCEDURES IN THE CRISIS DEVELOPMENT PROCESS’** highlights that in a crisis situation every option is being explored to delay the admission of people with dementia for as long as possible, including the use of psychotropic drugs. ‘Difficult’ cases are cared for in nursing homes for years, and every effort is made to avoid admission: *‘So we don’t give up straight away. We do reach our limits and complain and shout, but we’re not the kind of people who say: “Well, that’s it” and go straight in. So, we really try to find a solution, first call the PIA again, ask: What alternatives are there? What can we change about the medication? That doesn’t help any more. Make another change, talk to relatives again, get the care assistant on board so that we can look at this again. So, we are already looking at that. So, no, we’re actually a nursing home that doesn’t act so quickly’* (B14, pos. 148, nurse). The interviewees indicated that managers are key actors in the crisis management process. The way in which managers treat employees affects their well-being and ultimately also the level of stress caused by behaviour that challenges and referrals to geriatric psychiatry. They reported that managers try to minimise the burden on nursing staff, e.g. by admitting less complex residents to the nursing home or by not immediately filling vacant beds: *‘I’ve realised for myself that I always look at the teams a bit, […] if I have a bed free in the residential group, where it’s already very stressful anyway, then I try not to take on someone else […] where you already know at the beginning, ah yes, day-night rhythm disturbed […]. Then I would rather take someone else, where I know, ah, we can get away with it a bit easier. But I simply have to do that to keep the people in the team reasonably stable’* (B7, pos. 73, nursing home director).

The subcategory **‘INDIVIDUAL AND PSYCHOSOCIAL FACTORS’** shows that the resilience and personal attitude of nursing staff have an influence on the timing of referrals. According to the interviewees, some nursing staff are so overwhelmed by the behaviour that challenges of residents that it also places a burden on their private lives. With regard to nursing staff in nursing homes, there are also reports of overinterpretation of situations, difficulty in maintaining closeness and distance or a professional attitude, and a declining tolerance for frustration. The personal attitudes of GPs towards behaviour requiring treatment also play a role in referrals. The respondents emphasised that people with dementia react to the behaviour of nursing staff: *‘When a colleague comes in who has to work here at lunchtime and bangs things on the table somewhere or bangs her cup. Or she drops a tray because she’s not focussed. And they notice that too. And then they get more restless’* (B11, pos. 289, nurse).

Furthermore, the interviewees highlighted that referral to geriatric psychiatry occurs when nursing staff reach their breaking point in crisis situations and are no longer able to remain professional and respond to the needs of people with dementia. They reported that referral also occurs in cases of defensive behaviour on the part of residents and stressed staff. In addition, a higher sickness rate among nursing staff leads to a decline in motivation to provide care. They also mentioned that some nursing staff are afraid of residents and that admission to geriatric psychiatry represents a burden relief for the nursing staff. Some interviewees also pointed out that the breaking point varies from nursing home to nursing home. Restlessness and a tendency to run away are particularly difficult to cope with and lead to excessive demands and helplessness on the part of the staff. The behaviour that challenges of the resident then becomes unbearable for the nursing staff, crisis occurs, which is why a referral is made: ‘*So these are situations like, he doesn’t sleep at night, he’s very restless at night, and where you actually think, well, the day should be structured, then he’d be tired too. And then an admission is issued because […] everyone is at their wits‘ end, and the situation escalates more and more, and then an admission is made, which I don’t think is actually medically indicated, yes, but it is carried out (…) simply to relieve the situation, right’* (B15, pos. 133, GP).

It can also be inferred from the interviews that expectations regarding the success of treatment influence readmissions to geriatric psychiatry. The interviewees stated that treatment is successful if behaviour that challenges is reduced and the mood of people with dementia has improved or their quality of life is better. Persistent behaviour that challenges is often the reason for new escalating situations and readmission. The respondents also highlighted the importance of people with dementia being able to participate and cooperate in everyday life at the nursing home. If this is not the case, readmission is likely. They also acknowledged that unrealistic expectations of treatment success sometimes exist and that expectations vary between inexperienced and experienced nurses: *‘There is also this somewhat naive wish […] that he will now be admitted and come back like the phoenix rising from the ashes. And, yes, that’s just not the case. And it’s much easier to imagine that with inexperienced staff than with old hands, right’* (B10, pos. 65, psychiatrist).

## Discussion

In our qualitative study, we investigated the contextual factors associated with dementia crises and the related referral and admission processes to geriatric psychiatry of people with dementia and behaviour that challenges living in nursing homes. The results of the data analyses show that the contextual factors are highly complex. This extraordinary complexity of contextual factors is already evident in the multifaceted nature of dementia and the behaviour that challenges themselves. This study shows that the type and severity of dementia, the intensity and frequency of behaviour that challenges and the associated impact, such as endangering fellow residents and staff and increasing their stress levels, lead to escalating situations and is associated with admission to geriatric psychiatry. The Australian study by Djekovic et al. [45] also shows that the severity of behaviour that challenges is related to admission to hospital. It found that almost half of dementia patients in specialised dementia care units exhibited severe behaviour that challenges upon admission, and a large proportion of patients exhibited at least five behaviours. Hessler et al. [46] highlight in their German cross-sectional representative study in general hospitals that expansive symptoms such as aggression, irritability, nighttime disturbances, deviant motor behaviour and disinhibition are a burden on nursing staff and associated with many complications.

This clearly shows that the complex and multifaceted nature of dementia and behaviour that challenges in different countries and settings has a significant impact on care practices. This complexity makes it considerably more difficult to develop and implement appropriate, effective interventions. The present study illustrates that implementing adequate interventions is difficult due to the multifaceted nature of behaviour that challenges. In addition to identifying the causes, both non-pharmacological and pharmacological measures are used. However, these measures are often not effective in the long term, which is why crises occur and people with dementia are referred to psychiatric wards. Thompson et al. [47] confirm in their systematic review that non-pharmacological measures such as music therapy and multisensory interventions only bring about inconsistent improvements in distress and well-being.

The multifactorial and multidimensional nature of dementia and behaviour that challenges, and the fact that there is little evidence for non-pharmacological measures, explain why there is currently no uniform, standardised approach to dealing with this behaviour that is actually feasible in practice and has a lasting effect. Cho et al. [48] demonstrate that there are currently no clear guidelines on which non-pharmacological interventions are suitable for specific symptoms associated with behaviour that challenges. The effectiveness of such measures varies depending on the symptom, but overall, it is weak and inconsistent. Furthermore, the available evidence is rarely implemented in practice, mainly due to a lack of time and resources among nursing staff in long-term care facilities [48]. Nevertheless, this study shows that non-pharmacological measures, in particular needs-based care and relationship building, can play an important role in dementia care. When implementing non-pharmacological measures, it becomes clear that requirement to provide one-to-one care, person-centred communication and day structuring activities often cannot be met in practice.

The qualitative study by Mlinar Reljić et al. [49] emphasises the importance of a holistic approach to care for people with dementia. They report that relatives want ‘*compassionate, loving, caring and dignified nursing care*’ for their family members with dementia and that their spiritual needs be considered [49]. When we compare these results with the present study, we see a discrepancy between the expectations of relatives regarding good dementia care and the lack of feasibility of non-pharmacological measures in everyday care due to the existing contextual factors, e.g. lack of resources.

Moreover, this study identifies structural conditions as a contextual factor that is associated with the ability of healthcare professionals to act and can ultimately facilitate the referral of people with dementia. The current working conditions under which the healthcare professionals at the interface between geriatric psychiatry and nursing homes work on a daily basis make the adequate care of people with dementia considerably more difficult and further complicate the treatment barriers. Special circumstances, such as the COVID-19 pandemic, clearly demonstrated that treatment barriers were made even more challenging. Wong et al. [50] confirm in their qualitative study in Canadian nursing homes that staff shortages are a major barrier to adequate patient care. Oldenburger et al. [51] emphasise that the challenges in terms of staffing and time expenditure have been further exacerbated by the outbreak of the coronavirus.

In their descriptive qualitative study conducted in an Irish residential care centre, Connolly et al. [52] report on the profound stress experienced by residents and staff as a result of COVID-19 restrictions. They report that residents and staff felt disconnected from reality, from their families and also from other people within the facility with whom they had previously had contact [52].

The present findings underscore the discrepancy between existing care conditions, and the actual care needs of people with dementia and behaviour that challenges. In addition, the extent of previous Treatment barriers becomes clear when special circumstances arise, such as a COVID-19 pandemic.

The results of the present study underscore the importance of interprofessional cooperation as a key factor in cross-sector care between nursing homes and geriatric psychiatry. A lack of coordination in this area such as inadequate communication and deficient transitions from psychiatric care to nursing homes contribute to potentially avoidable readmissions. There were reports of insufficient information in discharge letters regarding changes in medication or diagnoses. The inadequate transfer of information in dementia patients is also evident in somatic hospitals. Gilmore-Bykovskyi et al. [53] found in their multi-site retrospective cohort study that discharge reports from somatic hospitals rarely systematically address psychological symptoms such as aggression, anxiety, risk of falling and their treatment. The use of psychotropic drugs is also only documented for a small proportion of patients [53]. Although the discharge reports show differences in content between psychiatric and somatic clinics, it is clear that the transfer of relevant information essential for post-care is generally inadequate. Moreover, the present study shows that the lack of information from somatic hospitals poses challenges for the care of people with dementia in nursing homes, which can then lead to admission to a psychiatric ward.

Furthermore, the results of this study show that there is a lack of necessary professional and environment-related resources at the interface between geriatric psychiatry and nursing homes to meet the complex care needs of this vulnerable patient group. These resources do not refer to the mere quantity of nursing staff and physicians available or the mere presence of materials and aids in the environment of people with dementia. Rather, it is about the quality of these resources, the expertise, practical experience and problem-solving skills of staff, continuity of relationships with people with dementia, the creation of a comfortable atmosphere, consideration of residents’ routines and the necessary specialisation in dementia care. The present study suggests that poor environmental management and a lack of daily structure contribute to permanent sensory overload and hinder the establishment of a supportive atmosphere for both residents and staff. Studies have shown that an environment tailored to people with dementia has a positive influence on them. Backman et al. [54] demonstrate in their scoping review that targeted environmental modifications for older nursing home residents (including those with dementia) are associated with significant improvements in emotional well-being and a reduction in behaviour that challenges. 

These findings underscore the importance of environmental context factors in the development and management of behaviour that challenges and the associated admissions to hospital.

With regard to professional resources, the present study shows that expertise and experience play a role in referrals to geriatric psychiatry and that less experienced staff may refer patients to geriatric psychiatry more quickly due to a lack of experience in resolving crisis situations. Wong et al. [50] point out that insufficient knowledge about recognising the side effects of psychotropic drugs, a lack of training in the use of non-drug interventions, and a reliance on psychotropic drugs when dealing with behaviour that challenges in nursing homes are key barriers. Duffy et al. [55] emphasise that gerontological expertise is a key prerequisite for nursing professionals to be able to provide people with dementia with needs-based, safe and high-quality care.

These factors contribute to critical situations becoming increasingly uncontrollable, stress levels rising and the system reaching its breaking point. This study suggests that managers play an important role in the crisis management process and that staff resilience and attitude are key factors in referrals to geriatric psychiatry. The degree of burden on nursing staff correlates with the behaviour that challenges. In particular, dysphoria/depression, disinhibition, restlessness and subjective mood influence the degree of burden [56]. Further studies show that managers’ leadership has a significant influence on the job strain of nurses [39] the implementation of person-centred care and the psychosocial climate in care facilities [40].

The present study also shows that the resilience, the personal attitudes and professional skills of nursing staff can influence the timing of a referral. The experiences, perceptions and perspectives of healthcare professionals show that referrals occur when nursing staff are overwhelmed by behaviour that challenges. Although Bretschneider et al. [57] do not specifically address the issue of being overwhelmed by behaviour that challenges in relation to referrals, they show that nurses’ inadequate management of changes in residents’ health status is associated with emergency situations and hospitalisations.

### Strengths and limitations

The study benefited from conducting individual interviews in the familiar surroundings of the participants, which enabled open and undisturbed communication. The decision not to use focus groups reduced group effects and ensured individual perspectives. To avoid bias, only people who were not known to the interviewer either privately or professionally were interviewed. The qualitative data analysis was carried out in two steps: First, the coding was done by one researcher (IHR), then it was discussed with a second researcher (VFU), and the category system was revised accordingly. Finally, the results were confirmed in a communicative validation process with healthcare professionals.

A key limitation is the limited transferability of the findings to other countries and regions, as the study focuses on the interface between geriatric psychiatry and nursing homes in rural areas in Germany. The legal basis for psychiatric admissions under the Mental Health Act varies between federal states and, in some cases, even between districts. There is no uniform regulation governing cooperation between nursing homes, GPs and clinics. Compared to countries with comprehensive mobile crisis services, gerontopsychiatric care in Germany is more inpatient-oriented. Nursing homes are also not required to have gerontopsychiatric expertise, so that relevant qualifications, e.g. in palliative care or geriatric psychiatry, vary considerably between regions and countries. The results can therefore only be transferred to other countries or healthcare systems to a limited extent.

In addition, the low participation intent of GPs, specialists and nursing homes with high rates of referrals meant that healthcare professionals who referred patients less frequently were interviewed, resulting in a potential sampling bias. These limitations should be considered when interpreting the results.

## Conclusions

While previous research has examined characteristics, reasons or contextual factors for admissions to general hospitals or has not specified the type of hospital in detail [15, 18, 58], our study specifically examines the interface between geriatric psychiatry and nursing homes. In particular, we focus on the contextual factors associated with the onset and management of dementia crises which are related to the referral and admission to geriatric psychiatry of people with dementia and behaviour that challenges.

The results show that the development of dementia crises and decisions to refer people with dementia to geriatric psychiatry are associated with contextual factors (Multifactoriality and multidimensionality of dementia and behaviour that challenges interventions in behaviour that challenges, structure-based ability to act, interprofessional cooperation, resources of the setting and coping with the crisis situation). The category ‘interprofessional cooperation’ shows that communication breakdowns at interfaces, unclear responsibilities, hierarchical role behaviour and the lack of an integrated and consistent approach to prevention, treatment and care for behaviour that challenges are relevant. These findings underscore the need for interprofessional, feasible interventions at the interface between geriatric psychiatry and nursing homes that remove structural barriers, improve institutional cooperation and promote the decision-making confidence of healthcare professionals in the crisis management process.

### Relevance to clinical practice

In order to better avoid psychiatric hospital admissions of people with dementia in challenging care situations in the future, structural, organisational and personnel changes at both the personal and professional levels are needed throughout the entire care pathway. First, outpatient geriatric psychiatric care must be expanded, for example through specialised mobile crisis teams (Dementia Rapid Response Teams) that can visit nursing homes at short notice, intervene on site and avoid admissions. At the same time, day clinics and outreach psychiatric care should be extended across the region in order to relieve the burden on inpatient resources in a more targeted manner and reduce waiting times. In nursing homes, staff shortages and legal restrictions on the scope of nursing care mean that in acute situations, action can only be taken when the relevant physician has issued the appropriate directive. The results indicate that nursing staff frequently encounter structural limitations in crisis situations due to their lack of legal authority to refer patients. To this end, it should be examined whether standardised crisis pathways could remedy this discrepancy. These crisis pathways could enable nursing staff to immediately call in medical support or specialised crisis services. Furthermore, there is a necessity to strengthen training in crisis intervention and interprofessional cooperation, thereby ensuring that teams are able to act in an emergency.

The results also show that interprofessional cooperation, especially cross-sector interprofessional communication, is inadequate. To improve interprofessional communication, we propose creating optimised structures for easier and faster information exchange (e.g. through digital solutions). When creating discharge letters and transfer forms, we suggest developing a standardised document that contains all relevant information for all professional groups involved. With regard to discharge, we recommend establishing standardised processes that prepare nursing homes and further care providers for discharge in good time before discharge in order to avoid ‘bloody’ discharges and associated readmissions. In cross-sectoral collaboration, we consider it very important to create low-threshold communication structures and to clarify expectations regarding treatment in geriatric psychiatry and the feasibility of treatment. This transparency in communication is also necessary when it comes to assessing the feasibility of dealing with people with dementia and behaviour that challenges in nursing homes. This is to avoid people with dementia being discharged to nursing homes that are unable to provide the complex care required by people with dementia and behaviour that challenges.

Moreover, the results of this study illustrate that unclear responsibilities and hierarchical role behaviour hinder the adequate care of people with dementia. To this end, we recommend the development of evidence-based, interprofessional treatment pathways with clear responsibilities for people with dementia and behaviour that challenges in order to promote standardised decision-making processes. The results also show the need for aftercare services. It would be helpful if the transition of care after hospitalisation were supported by structured aftercare services, e.g. through temporary outreach aftercare (psychiatric treatment at home), in order to avoid readmissions. Furthermore, the results show that some facilities lack specialisation in dementia. We therefore recommend structural changes in the nursing homes themselves, more dementia-friendly architecture and continuous training of staff in evidence based, effective interventions for behaviour that challenges and the prevention and management of dementia crises. In addition, the results illustrate that nursing staff are overwhelmed when dealing with behaviour that challenges and that their frustration tolerance is decreasing. To promote the psychosocial health of nursing staff, we suggest resilience-building team programmes and/or supervision. Overall, our research provides only a small insight into the care needs of people with dementia and behaviour that challenges. In many areas, there is a need for action in practical care that cannot be covered by a single measure. Rather, it is necessary to develop a coordinated, multidimensional package of measures in the sense of a complex interface intervention based on further research that addresses structural, interprofessional, and psychosocial levels.

### Implications for further research

The results of this study provide indications of how certain chains of factors contribute to the emergence of crisis situations. These considerations will contribute to further research by the lead author on the development of a model of assumed causal relationships, which should be verified in the further course of quantitative research.

Furthermore, quantitative approaches could help to systematically examine the contextual factors identified in this study and their interactions, and to quantify their relevance or frequency. However, quantitative research designs are necessary to prove the effectiveness of these presumed effects, e.g., hypothesis testing using structural equation modelling. Based on the effects then investigated, a complex interface intervention can be developed, which should then be further researched in RCTs for its effectiveness and reduction of readmissions.

## Supplementary Information

Below is the link to the electronic supplementary material.

Supplementary Material 1

Supplementary Material 2

## Data Availability

The data used in this study are included in the manuscript and supplementary materials. The complete interview data are not publicly available for data protection reasons. Further aggregated or anonymised data or coded excerpts can be requested from the corresponding author.

## Abbreviations

APN: Advanced Practice Nurse
BPSD: Behavioural and Psychological Symptoms of Dementia
COREQ: Consolidated Criteria for Reporting Qualitative Research
DGP: Deutsche Gesellschaft für Pflegewissenschaft (German Society for Nursing Science)
DRKS: Deutsches Register Klinischer Studien (German Clinical Trials Register)
GP: General practitioner
IHR: Imane Henni Rached
PCI: Problem-centred interview
PIA: Psychiatrische Institutsambulanz (Psychiatric outpatient clinic)
VFU: Victoria-Fabiola Ullmer
