# Supplementary material for: Healthcare professionals’ perspectives on contextual factors related to (re)referral and (re)admission to geriatric psychiatry of people with dementia and behaviour that challenges living in nursing homes in Germany: a qualitative study
Source: BMC Nurs. 2025 Dec 23;24:1488. doi: 10.1186/s12912-025-04117-2 (PMC12723930; doi:10.1186/s12912-025-04117-2)
Supplement: Supplementary file 1 — Supplementary Material 1 [file 12912_2025_4117_MOESM1_ESM.docx]

Table S1 Interview guide on contextual factors associated with dementia crises and the related referral and admission to geriatric psychiatry of people with dementia and behaviour that challenges living in nursing homes

| **PART 1: MULTIFACTORIALITY AND MULTIDIMENSIONALITY OF DEMENTIA AND BEHAVIOUR THAT CHALLENGES** | | | |
| --- | --- | --- | --- |
| **Key question/request for narrative** | **Key points – mentioned by the interviewee? Opportunity to ask follow-up questions** | **Maintenance and control questions** | **Specific questions** |
| What experiences have you had in dealing with people with dementia and behaviour that challenges in nursing homes? | - Does behaviour affect care? - Are you overwhelmed when dealing with these behaviours? - Lack of time and human resources to respond to behaviour? | What else comes to mind? | - In your opinion, how does the behaviour that challenges of people with dementia affect the level of care and support required in nursing homes? - In your opinion, what behaviour or symptoms lead to people with dementia being admitted to geriatric psychiatry? - In your opinion, what are the main factors that lead to admission, apart from the behaviour that challenges of people with dementia? |
| **PART 2: INTERVENTIONS IN BEHAVIOUR THAT CHALLENGES** | | | |
| **Key question/request for narrative** | **Key points – mentioned by the respondent? Opportunity to ask follow-up questions** | **Maintenance and control questions** | **Specific questions** |
| In your opinion, how is behaviour that challenges in people with dementia prevented and managed in nursing homes? | - Primary care? Targeted 1:1 contact? - Orientation towards the resident's biography/resources in activities of daily living (ADL)? - Person-centred communication (validation?) - Group activities/day-structuring activities | Can you give specific examples? | - What experience have you had in identifying the causes/triggers of behaviour that challenges? - What experience have you had with non-pharmacological measures in relation to behaviour that challenges? - How do you assess the use of psychotropic drugs for behaviour that challenges? What challenges arise when administering such drugs? |
| **PART 3: STRUCTURE-BASED ABILITY TO ACT** | | | |
| **Key question/request for narrative** | **Key points – mentioned by the respondent? Opportunity to ask follow-up questions** | **Maintenance and control questions** | **Specific questions** |
| Can you describe situations in which you felt helpless/powerless in relation to people with dementia and behaviour that challenges? | - Long waiting times for treatment places? - Overwhelmed by caring for people with dementia and behaviour that challenges? Too few staff? - Difficulties in diagnosing and treating the causes of behaviour that challenges (delirium, UTIs, pain) in the local nursing home? - No adequate dementia care concepts? | How exactly did this manifest itself? | - How do you experience the transition of people with dementia and behaviour that challenges from nursing homes to psychiatric wards and vice versa (i.e. admission and discharge)? Where are the biggest gaps or deficits? - Can you describe situations in which a person with dementia was readmitted shortly after "successful" treatment in geriatric psychiatry? What exactly contributed to the fact that it was not possible for them to remain in the nursing home? |
| **PART 4: INTERPROFESSIONAL COOPERATION** | | | |
| **Key question/request for narrative** | **Key points – mentioned by the respondent? Opportunity to ask follow-up questions** | **Maintenance and control questions** | **Specific questions** |
| How would you rate the cooperation and communication between the various departments/professional groups (geriatric psychiatry/nursing home/general practitioner/specialist) with regard to people with dementia and behaviour that challenges? | - Lack of communication? Does this result in additional work? - Lack of knowledge about each other's working methods? - Insufficient formal communication (referral forms, discharge letters, transfer forms) leading to interruptions in care? | What else comes to mind? | - What disruptive factors/obstacles/problems exist in interdisciplinary cooperation and communication between the various specialist areas/professional groups (geriatric psychiatry/nursing home/general practitioner/specialist) with regard to admission to geriatric psychiatry or discharge to a nursing home? |
| **PART 5: COPING WITH THE CRISIS SITUATION** | | | |
| **Key question/request for narrative** | **Key points – mentioned by the respondent? Opportunity for follow-up questions** | **Maintenance and control questions** | **Specific questions** |
| In your opinion, when has the point been reached where it is no longer possible to care for people with dementia who exhibit behaviour that challenges in a nursing home and admission to a geriatric psychiatric ward is necessary? | - Worsening of dementia symptoms? - Intensification of behaviour that challenges 🡪 Escalation of the crisis? - Higher care requirements and excessive demands on nursing home staff? - Helplessness/powerlessness in coping with crisis situations related to behaviour that challenges? | Can you provide more details? | - Can you describe situations in which different departments/professional groups (geriatric psychiatry/nursing home/general practitioner/specialist) had different perceptions/opinions about what a person with dementia needs or when behaviour needs to be treated? - How would you rate the level of expertise on dementia and behaviour that challenges in relation to the decision to refer someone to a geriatric psychiatric ward? Can you describe situations in which people with dementia and behaviour that challenges were more likely to be referred to a geriatric psychiatric ward due to a lack of specialist knowledge? - Can you report situations in which your personal stress (including private stress) or that of other staff members influenced the decision to refer someone? What factors influenced this? - What experiences have you had with people with dementia and behaviour that challenges during the coronavirus pandemic? To what extent do you think COVID-19 has influenced referral and admission to geriatric psychiatry? |
| **PART 6: RESOURCES OF THE SETTINGS** | | | |
| **Key question/request for narrative** | **Key points – mentioned by the respondent? Opportunity for follow-up questions** | **Maintenance and control questions** | **Specific questions** |
| In your opinion**,** what influence does **the setting,** i.e. the environment, the staff working there, the procedures, etc. (nursing home/geriatric psychiatry) have on people with dementia and their behaviour? | - Limited time and human resources lead to behaviour that challenges, especially defensive behaviour (trigger) - Difficulties experienced by people with dementia in adapting to routines in nursing homes/geriatric psychiatric wards. - Unfamiliar surroundings lead to behaviour that challenges | What else comes to mind? | - What signs do you see that indicate that treatment in geriatric psychiatry has been successful? - Can you describe situations in which you considered referral/admission unnecessary/avoidable? - Can you report any situations in which relatives of residents influenced the decision to refer them? What factors influenced this? - Can you describe situations in which conditions in the nursing home influenced the decision to refer a resident? What factors influenced this decision? - Can you describe situations in which, for example, social conflicts or overworked nursing staff were medically justified reasons for referral to geriatric psychiatry? |
| - **Do you have any further comments?** | | | |
