# Supplementary material for: Healthcare professionals’ perspectives on contextual factors related to (re)referral and (re)admission to geriatric psychiatry of people with dementia and behaviour that challenges living in nursing homes in Germany: a qualitative study
Source: BMC Nurs. 2025 Dec 23;24:1488. doi: 10.1186/s12912-025-04117-2 (PMC12723930; doi:10.1186/s12912-025-04117-2)
Supplement: Supplementary file 2 — Supplementary Material 2 [file 12912_2025_4117_MOESM2_ESM.docx]

Table S2 Overview of the main categories (MC) and subcategories (SC), including usage, key quotations and coding frequency (CF)

| **MC 1: MULTIFACTORIALITY AND MULTIDIMENSIONALITY OF DEMENTIA AND BEHAVIOUR THAT CHALLENGES** | | | | |
| --- | --- | --- | --- | --- |
| ***SC*** | ***Usage*** | ***Text passages*** | | ***CF*** |
| ***1.1: CHARACTERISATION AND COMPLEXITY*** | Severity of dementia and the type and intensity of behaviour that challenges influence admission to psychiatry, especially aggression towards oneself or others, agitation and restlessness, shouting and screaming, rejection, other somatic diseases or psychiatric disorders which need regularly medications lead to admission | *‘What is always very difficult for us is aggressive behaviour. When a resident is auto aggressive but is also aggressive towards others..’* (B7, pos. 5, nursing home director)  *‘But of course with dementia there is also refusal to eat, refusal of food, refusal of fluids. If the patients then get into a state where they are no longer supplied. In fact, we have already had patients who refused to be washed, who then somehow, who simply no longer allowed themselves to be cared for, where we then also said, maybe it's time for geriatric psychiatry.’* (B15, pos.16, GP)  *‘Sometimes you reach your limits, depending on the severity of the challenging behaviour, right? Sometimes you're helpless and then you don't know yourself.’* (B14, pos. 3, nurse) | | 92 |
| ***SC*** | ***Usage*** | ***Text passages*** | | ***CF*** |
| ***1.2: IMPACTS*** | Serious effects that the behaviour can have on the person with dementia and others, the danger to themselves and others, the suffering and helplessness of people with dementia due to their illness, the high level of care required and the increased stress level for staff and other residents | *‘Yes, if it simply becomes too abusive or if it becomes too impulsive, then it is also a danger to the employees, yes. So, we also have to protect these employees. Or other residents as well.’* (B9, pos. 15, nursing home director)  *‘The staff also become more restless. They then work completely differently. They're somehow so agitated internally, so agitated. And of course that is then transferred to the other residents.’* (B4, pos. 7, nursing service manager) | | 86 |
| **MC 2: INTERVENTIONS IN BEHAVIOUR THAT CHALLENGES** | | | | |
| ***SC*** | ***Usage*** | ***Text passages*** | | ***CF*** |
| ***2.1: TRIGGER IDENTIFICATION*** | Lack of awareness/reflection of behaviour and situation, insufficient depth of behaviour and situation analysis, looking for a quick solution (admission to psychiatry as solution); (poor) focus on the life, biography and identity of the person with dementia; insufficient somatic cause clarification, especially pain | *'And then I always say: What has just triggered him? Because these are always moments when they get triggered. Did you go too fast? Did you not take enough time?’* (B11, pos. 187, nurse)  *‘And we have to fight hard to ensure that a somatic assessment is carried out first and that not everything is attributed to the mental illness in the first place. And it's urinary tract infections, pain and so on that tend to be forgotten, and then we have to take more medication or even more without considering the somatic component.’* (B10, pos. 49, psychiatrist) | | 73 |
| ***SC*** | ***Usage*** | ***Text passages*** | | ***CF*** |
| ***2.2: NON-PHARMACOLOGICAL MEASURES*** | Orientation towards physical/psychological needs and relationship work is very important, lack of attention leads to behaviour that challenges (e.g. aggression), 1-to-1 care often cannot be guaranteed; avoid identified triggers and overwhelming stimuli; person-centred communication; day structure and activity (lack of daily structure can lead to behaviour that challenges and admission); | *‘And just being with him sometimes. Sometimes all it takes is this appreciation, attention, a caress, a hug. Everything that a person actually needs. These needs. Sometimes I don't even have to tell them but just give them a hug. This feeling: Oh God, no, I'm being taken seriously, I'm being seen. I feel someone. [...] And you can actually achieve a lot with that, can't you?’* (B14, pos. 35, nurse).  *‘I have a resident situation in my head where a resident had reacted very aggressively to women, but totally/ you could get him down again when someone came in a uniform. We realised this because the staff had to call the police twice because he was so aggressive and was throwing all the furniture. And the moment a uniformed person stood in front of him, there was silence. So that's a familiar story. Or that it's the gender that triggers it. If someone reacts badly to a man, we try to only send women into care, and vice versa, yes.’* (B7, pos. 27, nursing home director) | | 87 |
| ***SC*** | ***Usage*** | ***Text passages*** | | ***CF*** |
| ***2.3: USE OF PSYCHOTROPIC DRUGS*** | (un)reflected and (un)targeted use, used for escalation prevention, benefit-risk assessment, missing evaluation for discontinuation, usage is dependent on nursing staff; it is difficult to administer the medication (oral intake, depot medication) – therefore minor effect; capacities exhausted - everything has been tried and found to be insufficiently effective, paradoxical effect; is used to manage the daily care routine, to increase the residents' ability to co-operate; acceptance of somatic and psychiatric side effects to do not disturb the environment or to reduce the suffering of people with dementia; | *‘And you don't always have to go straight to the on-demand medication to sedate the resident. I first have to find out the reason why the resident has displayed the challenging behaviour right now, right now. And often we can actually validate the resident's behaviour and pull them out and go for a walk rather than administering half a Tavor or something like that.’* (B12, pos. 31, nursing service manager)  *‘Well, I'll say, in our environment it's difficult to administer them because we can usually only give them orally.’* (B6, pos. 51, nurse)  *‘But that is hardly tolerable. There are also visitors, and if they are now defecating in the corridor or are really so aggressive, what should be done? Of course, he was also on a high dose of risperidone. So, I was really at the end of my tether.’*(B10, pos. 31, psychiatrist)  *‘It's necessary, there's no other way. But we are aware of all the advantages and disadvantages of administering medication in geriatrics.’* (B1, pos. 75, GP) | | 77 |
| **MC 3: STRUCTURE-BASED ABILITY TO ACT** | | | | |
| ***SC*** | ***Usage*** | ***Text passages*** | | ***CF*** |
| ***3.1: TREATMENT BARRIERS*** | Limited treatment options for behaviour that challenges in dementia, constant readmissions, not knowing what to do next, limited options in geriatric psychiatry, therefore discharged again on the justification of being out of therapy, no effective non-pharmacological treatments; diagnostic and therapeutic barriers through a lack of technical equipment, e.g. electrocardiogram machine in nursing homes, limited physician availability, lack of specification of dementia; regulatory hurdles in that nursing staff are not allowed to make referrals or prescribe medication, nothing works without a physician's order, many steps have to be taken before admission (police/supervisory authorities scrutinise admission); COVID-19 as an additional burden for care staff, relatives and people with dementia, requirements of health authorities (residents must wear masks), worsening of dementia and behaviour that challenges due to contact restrictions, isolation, admission made more difficult by conditions (negative tests etc.) - admission to geriatric psychiatry categorically rejected | *‘So, we still have this case at the moment. It will stay forever, that won't change. But this constant screaming is a burden on the residents. She's no longer admitted because she's completely out of therapy.’* (B16, pos. 39, nurse)  *'Sometimes we don't continue things because, for example, we can't write an electrocardiogram for a patient in a nursing home.’* (B15, pos. 99, GP)  *'So, the main problem [...] is this admission itself, because you have to take various steps first. That means I have to inform the public order office [German: Ordnungsamt] first. Then, if I'm lucky, they'll come early. Then I first have to explain: What's going on anyway? they then decide whether to make a referral or not? Or the police have to be called after all. That delays everything.’* (B14, pos. 47, nurse) | | 50 |
| ***SC*** | ***Usage*** | ***Text passages*** | | ***CF*** |
| ***3.2: CARE CONDITIONS*** | It is difficult to get someone admitted to the clinic, admission only in an emergency, elective admissions almost impossible, the clinic questions the necessity of admission; reduced bed capacity due to lack of staff in the geriatric psychiatry, therefore long waiting times; in all areas exist staff and time deficit (medical practices, nursing homes, geriatric psychiatry/clinics), overloading of the entire healthcare system, inadequate diagnostics due to lack of time and staff resources (physician), lack of care (example: one nurse for 45 residents) | *‘Most of the time we have the problem, and it's also clearly due to a lack of staff or capacity, that there's no space available in the [name clinic]. That's also a problem.’* (B14, pos. 47, nurse)  *'At night, in the nursing homes where I've worked so far, there's a registered geriatric nurse with [...] almost 45 patients and when you imagine that she starts washing at the front, there are patients with dementia, there are patients who can't go to the toilet themselves. And then she starts at the front and finishes at the back. And if one of the patients then somehow gets in the way, that's not possible in a nursing home. There are no staff resources to deal with that.’* (B15, pos. 8, GP)  *‘And of course you have to be honest and say that some of the challenging behaviour is of course also the result of staff shortages’* (B10, pos. 75, psychiatrist) | | 68 |
| **MC 4: INTERPROFESSIONAL COOPERATION** | | | | |
| ***SC*** | ***Usage*** | | ***Text passages*** | ***CF*** |
| ***4.1: INFORMATION EXCHANGE AND DECISION-MAKING*** | Inadequate information transfer, missing information at handover/in the discharge letter, no handover forms at discharge, background information on medication changes after discharge from geriatric psychiatry not comprehensible, psychosocial measures not comprehensible, information not transparent - diagnosis, handover forms and physicians' reports are not comprehensible, no information from geriatric psychiatry to psychiatric institute outpatient service (German: Psychiatrische Institutsambulanz, PIA) (discharge), no discharge report to PIA, higher workload due to queries; deficient communication between professional groups and facilities when changing medication, lack of communication between nursing home and geriatric psychiatry - only shortly before discharge, lack of communication between GPs/pharmacy - several medication plans --> nursing staff do not know what to follow, insufficient communication between nursing home and nursing home/PIA and PIA, geriatric psychiatry and PIA; information is not reliable, information is glossed over, data is outdated or not up to date, relatives conceal dementia diagnoses and behaviour that challenges, information from somatic hospitals/geriatric psychiatry is concealed; questioning hospitalisation, questioning the competence of other professional groups, different opinions about when admission is necessary, psychiatrist does not trust the GP's judgement regarding the need for admission - nor does the outpatient specialist, additional challenge if patient does not show the behaviour that challenges (to the same extent) in the clinical environment - questioning the need for admission on the part of psychiatry, different opinions among nurses in nursing homes about the need for admission | | *‘Well, what I experienced again and again is that information was only sparsely available. That [...] the triggering situations were not really described or communicated. Yes. That is the main problem, [...] that it must be researched.’* (B2, pos. 60, psychiatrist)  *‘Sometimes these inaccurate statements from geriatric psychiatry. […] I experienced a situation where a care nurse really wanted to refer someone to us just because she liked them and felt sorry for them, but we said we couldn't take the person in because she had embellished it first. And only after we started to ask the right questions did we notice her behaviour: Oh, she wants to sugarcoat the fact that we're taking him and that won't work. So, we really need honesty here, because that's the prerequisite for dementia, because we can't look inside the resident.’* (B14, pos. 78, nurse) | 111 |
| ***SC*** | ***Usage*** | | ***Text passages*** | ***CF*** |
| ***4.2: ROLES AND COLLABORATION*** | Availability of the GP/psychiatric institute outpatient department is poor and waiting times are too long, the GP/PIA only comes to the nursing home after weeks, running after the GP because of referrals, GP is not always available at weekends and the on-call physician is ‘unsuitable’ for dementia (lack of specialist knowledge), GP cannot reach a GP for consultation, GP very rarely visits nursing homes for financial reasons, discharge/admission to nursing home only on Wednesdays or Fridays due to GP's availability, resident's general condition has already deteriorated by the time the physician arrives at the nursing home; role allocation and hierarchies; unclear allocation of roles and hierarchies hinder patient care, GP discontinues medication prescribed by a specialist, responsibilities for dementia unclear, the physician decides on referrals and not the nursing staff, geriatric specialist nurses are degraded in their competence, responsibilities between physicians unclear, who is responsible for referrals, disagreement between physicians regarding medication; lack of knowledge of each other's working methods (professional groups and settings), no interprofessional perspective, GP has a different focus (physical problems) - discrepancy between psychiatric and somatic treatment, GP has a time-shifted perception (e.g. GP does not mind if patients are restless at night because he is not present) | | *‘When he (the resident) comes for the first time, we also make sure that we don't do this admission on certain days, i.e. not on Wednesdays or Fridays, because we always have to make sure that the physicians are available when, let's say, the familiarisation period lasts a little longer. That's another important point.’* (B12, pos. 55, nursing service manager)  *'Competence dispute, GP says he's better than the specialist: “We can sort it out here too.” The specialist in turn says: "That's no longer our job, please let the GP sort it out." The nurse says: "You both have no idea. I'm here 24/7, you haven't got a clue, why don't you listen to me?" So, it's a three-way relationship.’* (B13, pos. 83, nursing service manager) | 76 |
| ***SC*** | ***Usage*** | | ***Text passages*** | ***CF*** |
| ***SC 4.3: TRANSITIONS*** | Outpatient services are perceived as a burden relief, faster solution and admission with PIA, specialist (PIA) is called in for excessive demands - also for referral to psychiatry, if no PIA is involved, this is a burden, first measure for behaviour that challenges is to involve PIA for referral, early consultation of GP for referral to PIA to be able to intervene early and avoid crises, long waiting times for PIA connection, PIA limited in its resources, inadequate neurological care in nursing homes; Discharge not announced in good time, too little medication handed out, faster discharge to nursing home as aftercare is guaranteed, desire for aftercare by PIA/specialists, on-site assessment of patients in psychiatry before admission to nursing home/discharge attempt in nursing homes seen as helpful; | | *‘We then took in a woman with severe dementia here. She was only ever treated by her GP. At some point, it just didn't work anymore. And the GP issued referrals. It took us nine months until the PIA was here. […] And if we can't bridge that [...] with a GP, then we have a problem here.’* (B17, pos. 111, nursing service manager)  *‘You also have to admit that there are also many so-called “bloody” discharges [...] It's not the case that people, if they're good, are often still there for a long time, but then it's more or less stopped and then [...] they're put back into the nursing home, because you know they're well looked after. ’* (B10, pos. 45, psychiatrist)  *‘I miss the aftercare, that you always have someone on site or that someone comes round after two or three days or after a week and says: “And how is it with the resident? Is it going well? Is everything all right?”’*(B14, pos. 53, nurse) | 39 |
| **MC 5: RESOURCES OF THE SETTING** | | | | |
| ***SC*** | ***Usage*** | | ***Text passages*** | ***CF*** |
| ***5.1: PROFESSIONAL FACTORS*** | Staff's lack of language skills leads to behaviour that challenges, different understanding of care by international staff; nursing staff in geriatric psychiatry are seen as specialists, little specialist knowledge/little practical experience leads to referral to psychiatry, education can also be a hindrance – ‘overcare’ by psychiatric nurses (every behaviour is seen as a symptom or impairment), lack of reflection on care situations leads to referral because it is not considered individually but a standard approach is taken; lack of experiential knowledge of new employees in the facility, professional experience is perceived as a resource when nursing home staff grow old, remain capable of acting in difficult situations, experienced nursing staff = preventive measure; lack of relationship building/staff changes makes care more difficult, continuity and relationship has an effect on the behaviour of the resident, patient shows the same behaviour in the nursing home after a stay in geriatric psychiatry (nurse triggers - negative relationship); care assistants/volunteers as a great burden relief, they are responsible for day structuring activities/therapeutic measures, as the nursing staff cannot do this due to other demands; relatives as a resource/burden relief but also as a trigger/burden, palliative decisions made more difficult due to relatives' lack of knowledge, nursing home must meet the demands of relatives regarding the care of residents (time-consuming), relatives as support for admission (contacting the physician), delayed/avoided admission because relatives do not know what happens in psychiatry | | *‘We have more and more nursing staff […] with a migration background. Language is often a barrier. Employees with a migrant background bring their culture and their understanding of the profession with them. They do not always fit in with that, so they are not always congruent.’* (B17, pos. 9, nursing service manager)  *‘So, I would say that there is an absolute correlation between the lower the level of expertise, the quicker [...] the desire to refer patients, of course.’* (B10, pos. 65, psychiatrist)  *‘I would say that this has happened due to employees who were new to our nursing home. Who simply experienced things differently and acted differently beforehand. Who didn't intervene and evaluate in advance, who were simply on day shift. And they couldn't cope with it.’* (B13, pos. 121, nursing service manager) | 130 |
| ***SC*** | ***Usage*** | | ***Text passages*** | ***CF*** |
| ***5.2: ENVIRONMENTAL FACTORS*** | Environmental barriers (room size, angles, light, odours), atmosphere of the ward/residential unit is important, - security, well-being, feeling at home, no possibility to retreat; more and more stimuli (sensory overload), which has an impact on staff and residents, rapid care procedures due to staff shortages, staff resources are needed to create a daily structure, no individual care possible due to rigid shift schedules and daily routine structuring, hectic pace leads to behaviour that challenges, disregard for the routines of people with dementia is a trigger for behaviour that challenges, routines in the nursing home (rules regarding order and cleanliness) cause stress; unprotected residential care unit leads to referral to geriatric psychiatry, frequent reason for readmission if wandering tendencies cannot be controlled, nursing home is too unsafe for residents, referral is used to change living arrangements, residents with wandering tendencies are sedated with medication to prevent them from leaving; lack of dementia specialisation (no dementia concept/no separate dementia residential area); hospital admissions are a disaster for people with dementia because of the change of environment and the consequences (deterioration of cognition/activities of daily lives), different behaviours in different settings, changed environment in the psychiatric ward leads to the disappearance of behaviours that reappear after discharge in the nursing home, problems that did not show up in geriatric psychiatry remain in the nursing home despite successful psychiatric treatment, change of environment leads to readmissions, people with dementia need time to adapt to the new environment (acclimatisation phase) | | *‘If I have enough resources, i.e. enough staff, to create a daily structure to make the patient somehow tired during the day. Or if they somehow don't understand or misunderstand something, to have a calming effect on them. If I have the resources for that, then the situations don't escalate.’* (B15, pos. 125, GP)  *‘We only have protected units, we don't have closed units. This means that we can't keep someone here who really tends to run away, who really wants to leave every minute, every two minutes. [...] And then we can't keep someone who is a danger to him or herself or to others, or lock rooms, for example. Five-point fixation, three-point, it doesn't matter. We can't do that here.’* (B13, pos. 164, nursing service manager) *‘The circumstances in the clinic differ from those in everyday life in a nursing home. And sometimes something that was perceived as a positive development in a clinic [...] was seen as an improvement that did not remain as constant in everyday life, but instead things that had previously been problematic reappeared, where there was supposedly no improvement at all.’* (B2, pos. 62, psychiatrist) | 108 |
| **MC 6: COPING WITH THE CRISIS SITUATION** | | | | |
| ***SC*** | ***Usage*** | | ***Text passages*** | ***CF*** |
| ***6.1: PROCEDURES IN THE CRISIS DEVELOPMENT PROCESS*** | All possibilities are exhausted to admit patients as late as possible (also psychotropic medication), ‘strenuous’ cases are also cared for in nursing homes for years and every effort is made to avoid admission; the way management treats staff has an impact on their well-being - and ultimately also on the overload caused by behaviour that challenges and referral to geriatric psychiatry, management tries to minimise the burden on nursing staff (takes on less complex residents, does not immediately reoccupy beds that have become free) | | *'So, we don't give up straight away. We do reach our limits and complain and shout, but we're not the kind of people who say: “Well, that's it” and go straight in. So, we really try to find a solution, first call the PIA again, ask: What alternatives are there? What can we change about the medication? That doesn't help any more. Make another change, talk to relatives again, get the care assistant on board so that we can look at this again. So, we are already looking at that. So, no, we're actually a nursing home that doesn't act so quickly.’*(B14, pos. 148, nurse)  *‘I've realised for myself that I always look at the teams a bit, [...] if I have a bed free in the residential group, where it's already very stressful anyway, then I try not to take on someone else [...] where you already know at the beginning, ah yes, day-night rhythm disturbed […]. Then I would rather take someone else, where I know, ah, we can get away with it a bit easier. But I simply have to do that to keep the people in the team reasonably stable.‘*(B7, pos. 73, nursing home director) | 41 |
| ***SC*** | ***Usage*** | | ***Text passages*** | ***CF*** |
| ***6.2: INDIVIDUAL AND PSYCHOSOCIAL FACTORS*** | Resilience and the personal attitude of staff have an influence on the timing of referrals, attitude of GPs towards behaviour requiring treatment, nurses overinterpret situations and referrals are made, nurses are overwhelmed by behaviour that challenges, behaviour is also a private burden, frustration tolerance decreases, people with dementia react to the behaviour of nursing staff with behaviour that challenges, difficulties in maintaining closeness and distance/maintaining a professional attitude; referral when the breaking point reached, care is at the limit, resources exhausted, no possibility of responding to the needs of people with dementia, referral to geriatric psychiatry also due to defensive behaviour (with stressed staff), higher sickness rate (decreasing care motivation), fear of patients, burden relief for nursing staff due to admission, more difficult handling of agitation/running tendencies leads to excessive demands/helplessness on the part of staff, reaching the breaking point varies from nursing home to nursing home; expectations of treatment success influence rereferral, treatment is successful when: behaviour that challenges is reduced/mood is improved/quality of life is better, consistent behaviour that challenges is often the reason for rereferral, ability of the person with dementia to cooperate/participate in everyday life, smooth everyday care, sometimes unrealistic expectations (different for inexperienced/experienced nurses) | | *‘When a colleague comes in who has to work here at lunchtime and bangs things on the table somewhere or bangs her cup. Or she drops a tray because she's not focussed. And they notice that too. And then they get more restless.’* (B11, pos. 289, nurse)  *‘So, these are situations like, he doesn't sleep at night, he's very restless at night, and where you actually think, well, the day should be structured, then he'd be tired too. And then an admission is issued because [...] everyone is at their wits‘ end, and the situation escalates more and more, and then an admission is made, which I don't think is actually medically indicated, yes, but it is carried out (...) simply to relieve the situation, right.’* (B15, pos. 133, GP)  *‘And [...] then there is also this somewhat naive wish […] that he will now be admitted and come back like the phoenix rising from the ashes. And, yes, that's just not the case. And it's much easier to imagine that with inexperienced staff than with old hands, right.’* (B10, pos. 65, psychiatrist) | 94 |
